# New Orders Affecting Service Nurses

**Published:** 1919-09-06

**Authors:** 


### New Orders Affecting Service Nurses.

The long-expected announcement has been made respecting war service gratuities to naval nursing sisters. They fall under three categories, members of Q.A.R.N.N.S., members of the Reserve and members of Voluntary Aid and Red Cross Association nurses. The minimum gratuity for Q.A.R.N.N.S. begins at £40 for sisters and superintending sisters, £45 for head sisters. In addition all who have completed more than one year of war service are entitled to £1 for every additional month's service (not exceeding 48) if outside the United Kingdom or in a hospital ship; for home service the monthly bonus is 10s. For members of the Reserve the gratuity is fixed at £30, and the bonus at 10s. a month irrespective of whether the service were at home or abroad. Voluntary Aid and Red Cross Association nurses are to receive a gratuity of £10 and 10s. per month extra. In all cases the awards are subject to satisfactory service having been rendered and will not be payable to members of Q.A.R.N.N.S. who resigned their appointment before November 11, 1918, after less than two years' service. War service reckons as the period between August 1914 and the termination of the war "as defined by statutory authority," on August 3, 1919. Applications should be addressed to the Accountant-General of the Navy, Admiralty, S.W. 1, except nurses still serving, respecting whom further instructions will be issued.

A new Army Order, issued August 28, provides for the granting of extra remuneration to nurses necessarily retained on military service. The weekly rate of the bonus for members of Q.A.I.M.N.S. and Reserve, and the T.F.N.S. is: staff-nurse 8s. 9d.; sister 10s. 6d.; assistant matron 17s. 6d.; principal matron 24s. 6d.; matron-in-chief 28s. In the military families' hospitals, charge nurses are to have 8s. 9d., and matrons 10s. 6d. V.A.D. nursing members are to receive 3s. 6d.; special military probationers 3s. 8d.; assistant nurses 5s. 3d. The bonus will only be issued subject to the nurse having signed to serve either as long as required or till April 30, 1920. Members of Q.A.I.M.N.S. and the permanent staff of military families' hospitals are exempt from this stipulation.

**Dentistry as a Profession**.—*Continued from* *p.* 560.

appointed a Departmental Committee to investigate the extent and gravity of the evils connected with the practice of dentistry and dental surgery by persons not qualified under the Dentists Act, to inquire into the expediency of legislation, and to consider and report. This report has now been published, and is most exhaustive in its nature. The chief recommendations put forward by the Committee are:

(i) An alteration of the law so as to secure the prohibition of the practice of dentistry by persons not registered.

(ii) The registration under certain conditions of unregistered practitioners practising dentistry at the date of our report.

(iii) A reduction in the minimum time required to be spent by dental students to acquire a qualification in dental surgery.

(iv) The provision of dental treatment for expectant mothers and children under the age of five years.

(v) The completion as rapidly as practicable of an adequate system of school dental treatment.

(vi) The establishment of a public dental service.

(vii) The employment of dental dressers or assistants acting under the supervision of registered dentists in school and public dental services.

(viii) The establishment of a system of scholarships for dental students with adequate maintenance grants.

(ix) The registration after a short course of study and examination of dental mechanics employed as such during five years before the date of our report.

(x) Scholarships for dental mechanics.

(xi) Increased grants to dental schools.

The first step for the dental student is to register with the General Medical Council, and it is strongly advisable that he should register both as a dental and as a medical student, in case he should wish to complete the curriculum for the conjoint diploma. Four years must elapse between this registration and the final examination for the L.D.S., and although training in dental mechanics and instruction in chemistry and practical chemistry and physics may be done before registration, the time spent at the work on these subjects will not be counted in the four years required unless registration precedes it. The recommendations adopted by the General Medical Council in 1909 as to the course of study and examinations are still under revision by the Council, and will doubtless be influenced by the attitude that Parliament adopts towards the recommendations of the Departmental Committee.

The necessary training in dental mechanics may be taken either in the mechanical department of a recognised dental hospital or with a registered practitioner, the first for preference. A Preliminary Science Examination has to be passed in chemistry and physics. The necessary study for this examination also counts as part of the four years of professional study if taken after registration, but not otherwise. This Preliminary Science Examination must be passed previous to the commencement of the two years' hospital course for the final examination. It is therefore advisable, if possible, to take the necessary classes for this purpose and to pass the examination during the first year of study.

When the student has passed his Preliminary Science, has attended lectures on dental mechanics and metallurgy, and completed the manufacture of the necessary dentures and crowns required by the curriculum, he will be in a position to pass the first professional examination. This consists of two parts: I. A practical examination in mechanical dentistry, conducted in the mechanical laboratory of one of the London dental hospitals; II. A written examination in dental metallurgy.

The second, or final, professional examination is in two parts. The first, in medical subjects, must be passed before the second, in dental subjects. It includes general anatomy and physiology, and general pathology and surgery. Part II. consists of dental anatomy and physiology, dental pathology and surgery, and practical dental surgery. These examinations are partly written and partly oral, and the student in offering himself must produce a certificate showing that he has attended lectures on anatomy and physiology, practical physiology, surgery, and medicine at a recognised medical school, and has performed dissections during not less than twelve months, and attended the practice of surgery and clinical lectures on surgery at a hospital or hospitals for twelve months during ordinary sessions. At the special dental part of the examination the oral examination is conducted by the use of preparations, casts, etc., and at the practical examination candidates may be examined (*a*) on the treatment of dental caries, on the filling of teeth with gold or other material, by inlaying or by crowning, and other operations in dental surgery; (*b*) on the treatment of the various irregularities of children's teeth. A certificate must have been produced showing that the student has attended at a dental hospital and school: (*a*) a course of dental anatomy and physiology; (*b*) a separate course of dental histology, including the preparation of microscopical sections; (*c*) a course of dental surgery; (*d*) a separate course of practical dental surgery; (*e*) a course of not less than five lectures on the surgery of the mouth which may form part of the course of lectures on surgery required for Part I. of the examination; (*f*) a course of dental bacteriology; (*g*) a course of dental materia medica; (*h*) a course of practical instruction in the administration of such anæsthetics as are in common use in dental surgery. A certificate must also be produced showing that the candidate has, during two years, attended the practice of dental surgery in a dental hospital or the dental department of a general hospital.

The cost of training varies slightly with the dental school, but can be roughly estimated at £200 for the two years' instruction in dental mechanics and the two years' hospital practice and lectures required by the Royal College of Surgeons. The schools in London are the Royal Dental, National Dental (University College Hospital Dental Department), Guy's Hospital, and the London Hospital. Most of the provincial universities offer dental training and grant degrees in dentistry.

The Royal Colleges of Surgeons of England, Glasgow, Edinburgh, and Ireland hold examinations for the L.D.S. and grant the diploma.

